# Large Protein Assemblies for High-Relaxivity Contrast Agents: The Case of Gadolinium-Labeled Asparaginase

**DOI:** 10.1021/acs.bioconjchem.2c00506

**Published:** 2022-12-02

**Authors:** Giulia Licciardi, Domenico Rizzo, Maria Salobehaj, Lara Massai, Andrea Geri, Luigi Messori, Enrico Ravera, Marco Fragai, Giacomo Parigi

**Affiliations:** † Magnetic Resonance Center (CERM), University of Florence, Via Luigi Sacconi 6, Sesto Fiorentino50019, Italy; ‡ Department of Chemistry “Ugo Schiff”, University of Florence, Via della Lastruccia 3, Sesto Fiorentino50019, Italy; § Consorzio Interuniversitario Risonanze Magnetiche Metallo Proteine (CIRMMP), Via Luigi Sacconi 6, Sesto Fiorentino50019, Italy

## Abstract

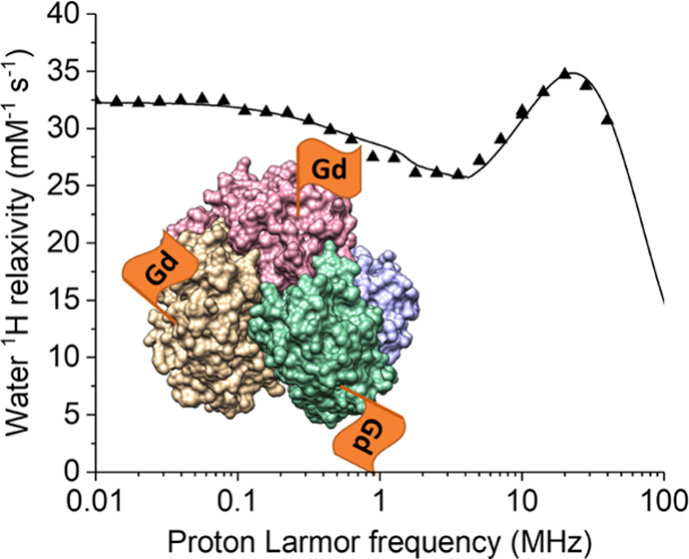

Biologics are emerging as the most important class of drugs and are used to treat a large variety of pathologies. Most of biologics are proteins administered in large amounts, either by intramuscular injection or by intravenous infusion. Asparaginase is a large tetrameric protein assembly, currently used against acute lymphoblastic leukemia. Here, a gadolinium(III)-DOTA derivative has been conjugated to asparaginase, and its relaxation properties have been investigated to assess its efficiency as a possible theranostic agent. The field-dependent ^1^H longitudinal relaxation measurements of water solutions of gadolinium(III)-labeled asparaginase indicate a very large increase in the relaxivity of this paramagnetic protein complex with respect to small gadolinium chelates, opening up the possibility of its use as an MRI contrast agent.

## Introduction

The millions of MRI exams performed annually in the world after administration of gadolinium(III) contrast agents and the concerns about their safety continuously stimulate the efforts for the development of safer contrast agents.^1^ The contrast agents used in the clinics are nowadays almost exclusively small gadolinium(III) complexes. Administration of these complexes has been shown to determine gadolinium accumulation in the tissues of the patients, thus raising concerns for their long-term consequences on health.^2,3^ For instance, they have been associated with nephrogenic systemic fibrosis in patients with impaired renal clearance.^4−6^

The strategy to reduce the risks associated with the administration of contrast agents without reducing the quality, and thus the diagnostic accuracy, of the MRI images, passes through the use of molecules not containing gadolinium(III) ions but similarly able to enhance the nuclear relaxation rates of water protons,^7−10^ or through the development of gadolinium complexes with higher efficiency so that the injected dose can be sizably reduced. A reduction of the injected gadolinium(III) dose can be achieved (i) by targeting the contrast agents to specifically accumulate them in the tissues of interest and (ii) by increasing the capability of the agents to enhance the water proton relaxation rates. This capability is called relaxivity and is defined as the enhancement in the water proton relaxation rate due to a gadolinium concentration of 0.001 mol dm^–3^.

An effective way to increase relaxivity at low and intermediate magnetic fields (below ca. 1 T) is slowing down the reorientation mobility of the complex. This can be achieved by functionalizing low-molecular-weight gadolinium(III) complexes to bind noncovalently to macromolecules,^11−13^ by confining them within nanosized matrices, like nanogels,^14−18^ or by exploiting nanosized gadolinium(III)-based compounds.^19−24^ On the other hand, an increase in the reorientation time of the contrast agent determines a decrease in relaxivity at high magnetic fields. The optimal reorientation time τ_*R*_ is related to the applied magnetic field *B*_0_ through the relationship τ_c_^opt^ = (γ_I_*B*_0_)^−1^, with τ_c_^–1^ = τ_R_^–1^ + τ_M_^–1^ + *R*_1e_, where γ_I_ is the proton magnetogyric ratio, τ_M_ is the lifetime of the water molecule(s) coordinated to the gadolinium(III) ion, and *R*_1e_ is the electron relaxation rate.^25,26^ At 1.5 T, if water exchange and electron relaxation are slower than molecular reorientation, τ_R_^opt^ = 2.5 ns. Therefore, reorientation times in the nanosecond timescale are needed to achieve the highest relaxivities at the fields of MRI scanners.

Metalloprotein-based contrast agents have been considered because of the ease of preparation and the availability of engineering techniques that can allow for their functionalization and targeting.^27^ The relaxivity of (either natural or metal-substituted) paramagnetic metalloproteins is however typically limited, despite their overall reorientation times of the order of nanoseconds or larger, due to the presence of paramagnetic ions (different from gadolinium) with short electron relaxation times, and/or the long lifetime of coordinated water molecules. Proteins engineered by rational design were thus proposed by creating a gadolinium-binding site with strong metal selectivity in a stable and potentially fully functioning host protein.^28,29^ Chimeric proteins were also constructed by inserting an EF-hand motif, which can bind a gadolinium ion, into a functionalized protein.^30−32^ However, the application of these chimeric proteins is limited by their metal binding affinities which are much weaker than those of the chelates approved for clinical use. A high binding affinity and metal selectivity was achieved by engineering an EF-hand motif of the protein α-parvalbumin.^33^

An easier, preferable alternative is attaching a paramagnetic tag to a diamagnetic protein, like albumin and immunoglobulins.^34^ Gadolinium(III) ions can be attached to proteins by bifunctional chelates, usually DOTA-like or DTPA-like complexes with an electrophilic group for conjugation to nucleophilic groups of macromolecules.^35^ The possibility of genetically engineering protein polymers at multiple backbone sites allows attaching multiple paramagnetic tags per protein.^36,37^ This results in multivalent, biomacromolecular contrast agents endowed with extremely high relaxivity per particle, although the relaxivity per gadolinium ion is often limited. GdDTPA-monoamide tethered to polylysine^38^ or dextran,^39^ for instance, shows low relaxivities because of the internal mobility which limits the relaxivity enhancement.^40^ Furthermore, an increase in the water lifetime of about a factor 3 has been observed upon the replacement of one carboxylate function by an amide in both DOTA-like and DTPA-like chelates.^41^

**Figure 1:**
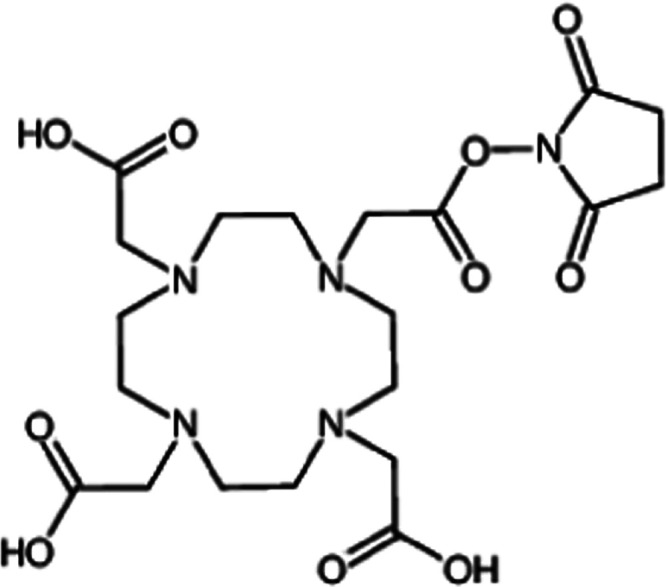
1,4,7,10-Tetraazacyclododecane-1,4,7,10-tetraacetic acid mono-*N*-hydroxysuccinimide ester (DOTA-NHS-ester) chemical structure.

Along these lines, we have attached a derivative of 1,4,7,10-tetraazacyclododecane-1,4,7,10-tetraacetic acid (DOTA) to the protein l-asparaginase II (ANSII), a biological drug in clinical use against leukemia. Together with the four macrocyclic nitrogen atoms, four acetate arms of the chelate coordinate the gadolinium(III) ion, and the carboxylate group on the 5-carbon arm, activated with the ester, is used for covalent attachment to the primary amine of lysine residues via amide bond formation (Figure 1).^35^ Carboxylates and backbone carbonyls, and hydroxyls to a lesser extent, can also coordinate lanthanoids.^42^ However, it has long been proven that in the presence of a high-affinity chelator and after thorough purification, the ions aspecifically bound to the protein surface are in negligible concentration.^43,44^ Analogous paramagnetic tags were previously attached to dendrimers, silica nanoparticles, or proteins like albumin through methanethiosulfonate anchor groups.^45^ In these cases, they showed a relaxivity not exceeding 25 s^–1^ mM^–1^ even at the peak magnetic field because of being limited by a subnanosecond reorientation time and a rather long lifetime of the coordinated water molecule.^45,46^

ANSII consists of four identical subunits, forming a dimer of dimers of 138 kDa with *D*_2_ symmetry, with an extensive mobility in the nanosecond timescale.^47^ The attachment of a paramagnetic tag to ANSII is thus expected to result in an MRI contrast agent of much improved relaxivity with respect to those presently used in clinics, and of the order of that of previously reported paramagnetic proteins. Here, the relaxivity properties of the Gd(III)-DOTA-NHS-ester conjugated to amine groups of ANSII have been investigated in detail to evaluate the relaxation enhancement achieved upon conjugation. Indeed, we found a relaxivity at 37 °C as large as ca. 35 s^–1^ mM^–1^ at the peak magnetic field.

Most importantly, this protein represents an attractive carrier for the delivery of the paramagnetic moiety because:

(1) ANSII is one of the oldest biologics approved for clinical use as a drug against acute lymphoblastic leukemia both in its native and PEGylated forms.^48^ It is currently used in humans by intramuscular injection or by intravenous infusion three times weekly. The grafting of gadolinium(III) tags is not expected to affect the therapeutic efficacy of ANSII as proved by the enzymatic activity of the clinically approved highly PEGylated form of the enzyme, where most of the surface exposed lysines are conjugated to PEG chains.^49^ Therefore, this protein is a good model to develop protein-based theranostic agents and also to develop new strategies to investigate the pharmacokinetics and fate of biologics.
(2) Each of the four ANSII subunits contains 22 lysine residues, which can be largely functionalized.^50^ The many solvent-exposed lysine residues can thus allow for the conjugation of a huge amount of paramagnetic chelates to the same protein. In perspective, this can allow the development of an agent carrying a high payload of paramagnetic ions.

## Results and Discussion

### Preparation and Characterization of the Conjugated Protein

The functionalization of ANSII with DOTA-NHS-ester was carried out as described in the Experimental Procedures section. Considering the lower p*K*_a_ of the α-amine of the N-terminus compared to the ε-amine of lysines, as a result of the inductive effects of the nearby carbonyl group, at pH 7.5, a selective acylation and alkylation of N-terminal amines is favored, although complete site specificity is not achieved.^51,52^ Under these conditions, a few amines in the protein are expected to be significantly reactive.^53,54^ Mild conditions for conjugation were employed, as we aimed at evaluating the paramagnetic relaxation enhancement with distant gadolinium(III) ions, to avoid possible magnetic coupling. Gadolinium(III) was added in defect, and the addition was followed by gel filtration purification. ICP-OES was employed to determine the gadolinium(III) concentration into the sample; it was found to correspond to the presence of 3 gadolinium(III) ions per tetramer.

### ESI MS Characterization of the Free and Conjugated Protein

**Figure 2:**
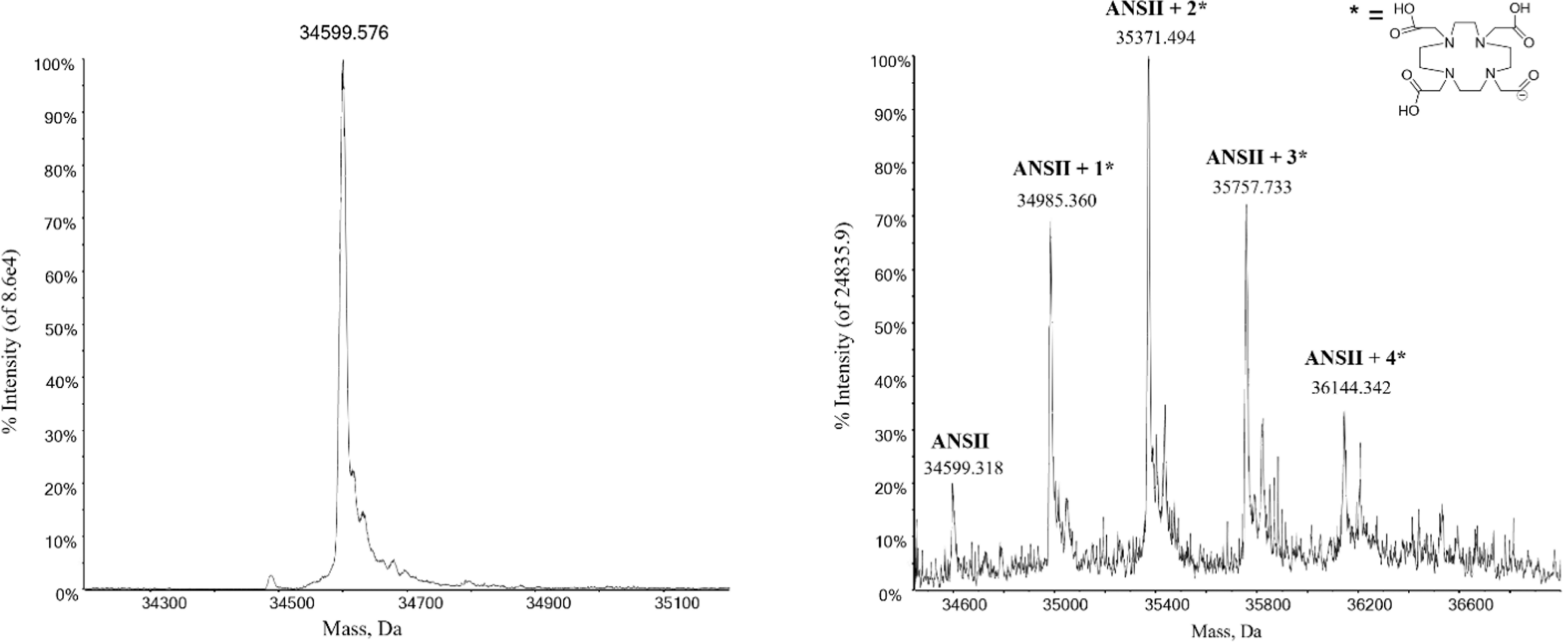
Deconvoluted mass spectra of ANSII 10^–6^ M (left panel) and deconvoluted mass spectra of DOTA-NHS conjugated protein 10^–6^ M (right panel).

The free and DOTA-NHS-ester conjugated protein were further characterized through ESI MS analysis according to standard procedures.^55,56^ The ESI MS spectrum of the free protein is shown in Figure 2a. The deconvoluted ESI MS spectrum shows an intense and very well resolved single peak with a mass of 34 599 Da. This value is very close—though not identical (there is an apparent difference of a few Daltons)—to the calculated value for the sequence of the protein reported in UNIPROT (P00805) being equal to 34 595 Da. Upon inspection of the ESI MS spectrum, it emerges that the ANSII protein shows a high degree of purity. The spectrum of the DOTA-NHS-ester conjugated protein, prepared as described in the Experimental Procedures section, is reported in Figure 2b. The latter spectrum shows a number of additional peaks with mass values greater than the free protein. Notably, the peaks at 34 985, 35 371, 35 757, and 36 144 Da are straightforwardly assigned to protein conjugates bearing one, two, three, and four DOTA-NHS-ester moieties, respectively. The percentage ratios between free ANSII and the various forms of ANSII conjugated with DOTA-NHS are reported in Figure S1. This means that the sample contains in comparable amounts a few species with a variable number of DOTA-NHS-ester groups. It is known that the DOTA-NHS-ester manifests a large selectivity for free amino groups, but it is difficult to identify by MS which lysine groups are actually modified. As only a rather limited number of lysine groups are modified even in the presence of relatively large DOTA-NHS-ester/protein ratios 15:1, it can be inferred that only the most accessible and reactive lysine groups will be modified. A plausible estimation of the most accessible and reactive lysines has been obtained independently through a bioinformatic analysis using the program PROPKA.^53,54^ This analysis suggests that the most reactive amino groups residues are the N-terminus and lysines 104, 49, and 71 (see Table S1).

### Water ^1^H NMRD Profiles of ANSII-DOTA

**Figure 3:**
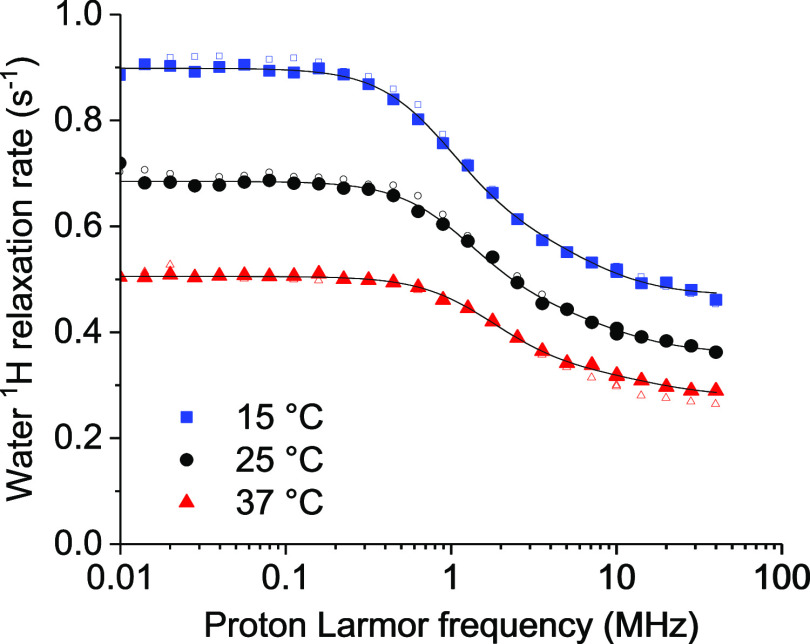
^1^H NMRD profiles of a water solution of DOTA-conjugated ANSII (0.38 mmol dm^–3^ monomeric protein concentration) at 15, 25, and 37 °C (solid symbols). Basically identical profiles were measured for the unconjugated ANSII protein (empty symbols).

The ^1^H NMRD profiles of water solutions of diamagnetic (metal free) ANSII-DOTA at 15, 25, and 37 °C are shown in Figure 3. The concentration of the monomeric protein was 0.38 mmol dm^–3^.

The decrease in the relaxation rates measured for increasing magnetic fields reports on the dynamics of the water protons interacting with the protein.^57^ Their dipole–dipole coupling energy is modulated by the shortest time between the overall reorientation time τ_R_ of the tetrameric protein assembly, the local internal mobility times τ_f_, and the lifetime τ_M_ of the water molecule on the protein surface. Water molecules with τ_M_ longer than τ_R_ or τ_f_ thus provide information on the molecular tumbling time and on possible presence of faster internal mobility. The ^1^H NMRD profiles were fitted using eq 1(58,59)
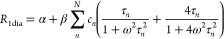
1where *c*_*n*_ are weight coefficients summing to 1. The profiles could not be nicely fitted with *N* = 1, whereas fits of good quality could be obtained with *N* = 2 (solid lines in Figure 3). The best-fit profiles are reported in Table 1, where the longest and shortest τ’s obtained from the fit were indicated as τ_R_ and τ_f_, respectively.^60^ These values are in agreement with those previously obtained for the unconjugated ANSII protein in a different concentration and water buffer solution (20 mm sodium phosphate, pH 7.5, 0.02% NaN_3_, 0.1 mg mL^–1^ protease inhibitors (Pefabloc)), when the following values were obtained at 25 °C: *c*_1_ = 0.42, τ_1_ = 6.0 × 10^–8^ s, τ_2_ = 9 × 10^–9^ s.^50^ The values of τ_R_ confirm that indeed the protein forms tetrameric assemblies, being of the order of what expected for globular proteins with MW of ca. 140 kDa.^58^ This finding represents an evidence that the tetrameric structure of the unconjugated protein is retained also upon its functionalization with DOTA-NHS-ester. The large contribution from correlation times smaller than 10 ns (at 25 °C) indicate the presence of an extensive mobility in the nanosecond timescale, probably reflecting the intrinsic flexibility related to the many loop regions present in the protein. As previously noted, this extensive internal mobility is consistent with the intensity and resolution of the solution NMR spectra of the protein.^50^

**Table 1:** Best-Fit Parameters of the ^1^H NMRD Profiles of DOTA-Conjugated ANSII

	15 °C	25 °C	37 °C	
α	0.47	0.36	0.28	s^–1^
β	1.2 × 10^7^			s^–2^
*c*_1_	0.31			
τ_R_ = τ_1_	8.7 × 10^–8^	6.7 × 10^–8^	4.9 × 10^–8^	s
τ_f_ = τ_2_^a^	1.3 × 10^–8^	9.3 × 10^–9^	5.5 × 10^–9^	s

a *c*_2_ = 1 – *c*_1_.

### Water ^1^H NMRD Profiles of Gd-DOTA-Conjugated ANSII

**Figure 4:**
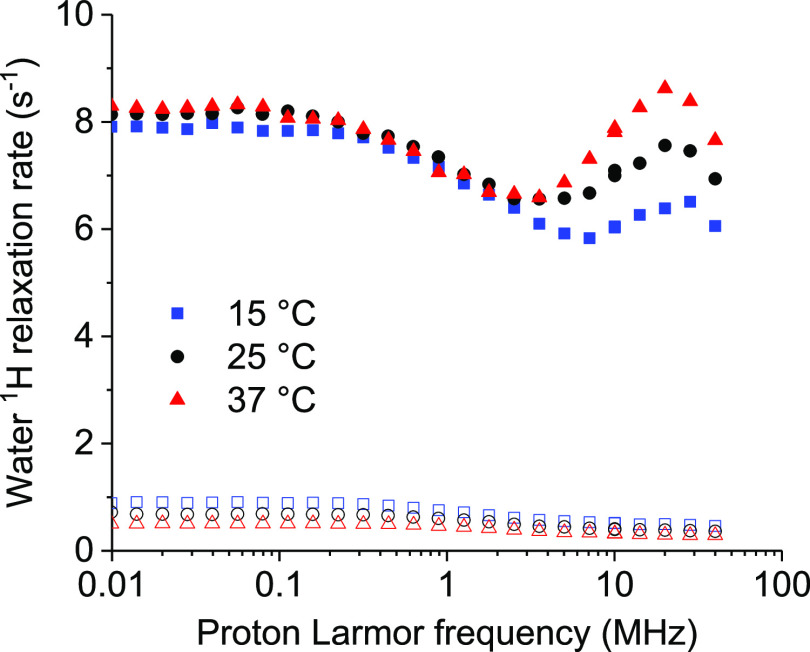
^1^H NMRD profiles of a water solution of Gd-DOTA-conjugated ANSII (0.24 mmol dm^–3^) at 15, 25, and 37 °C (solid symbols). The profiles of the Gd-free protein are also shown as empty symbols.

**Figure 5:**
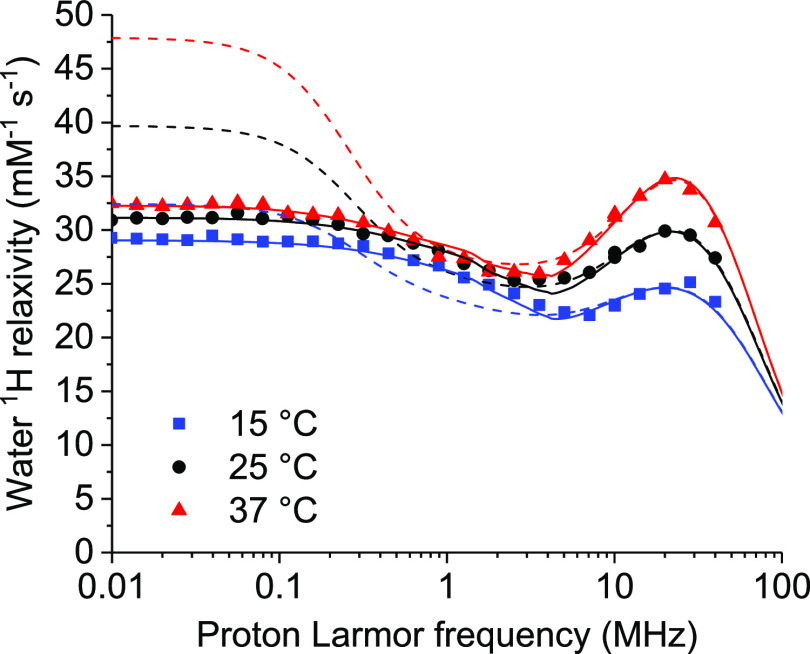
^1^H relaxivity profiles of GdDOTA-conjugated ANSII at 15, 25, and 37 °C. Solid lines are the best-fit profiles obtained with the Florence NMRD program, and dashed lines are calculated with the SBM model.

The NMRD profiles of the water solution of Gd-DOTA-conjugated ANSII were collected at 15, 25, and 37 °C (Figure 4). The concentration of the gadolinium(III) ions was 0.24 mmol dm^–3^, as determined from ICP-OES measurements. The relaxivity values, obtained from the difference between the relaxation rates measured from the paramagnetic and the diamagnetic samples, scaled to 1 mmol dm^–3^ gadolinium(III) concentration, are shown in Figure 5.

Interestingly, the relaxivity is quite large (although much smaller than the theoretical limit^61^) for a complex with one water molecule (*q* = 1) coordinated to the gadolinium ion, with respect to that measured for other proteins conjugated to DOTA-like or DTPA-like complexes. Nevertheless, the temperature dependence of the profiles indicates that the relaxivity is limited by the water protons exchange rate, as it increases with the temperature. The whole profiles cannot be fitted with the Solomon–Bloembergen–Morgan (SBM) model due to the presence of zero-field splitting (ZFS), which affects the energy of the electron spin states.^62,63^ The SBM model can however be used to reproduce the high-field regions of the profiles, when ZFS can be neglected because much smaller than the Zeeman energy. In the fit, outer-sphere contributions were also considered with typical values for the distance of closest approach and the diffusion coefficients.^64^ The fit of the high-field region (proton Larmor frequencies larger than 5 MHz) shows that reorientation times τ_i_ of the order of nanoseconds are needed to reproduce the profiles and their temperature dependence. These times are 1 order of magnitude smaller than the tumbling times of the tetrameric protein (τ_R_, see above), which implies that the dipole–dipole interactions between the unpaired electrons of the gadolinium(III) ions and the water protons are completely averaged out by internal dynamics.

The profiles were thus fitted using the modified Florence NMRD program,^63,65,66^ which can reproduce the effects of the ZFS both in the electron and the nuclear relaxation rates, in the Redfield and slow rotation limits.^67^ The slow rotation limit implies that reorientation is much slower than electron relaxation (determined from the parameters Δ_t_ and τ_v_ according to the pseudorotation model^68^). Contributions from even faster motions (with correlation time τ_l_) were included through a Lipari-Szabo model-free approach^11,69^ so as to further improve the quality of the fit. The best-fit parameters are reported in Table 2, and the corresponding profiles are shown in Figure 5 as solid lines. The dashed lines in Figure 5 show the relaxivity profiles calculated with the same parameters and using the SBM model.

**Table 2:** Best-Fit Parameters of the ^1^H Relaxivity Profiles of GdDOTA-Conjugated ANSII

	15 °C	25 °C	37 °C	
*r*^a^	3.05			Å
*q*^a^	1			
Δ_t_	0.0095			cm^–1^
τ_v_	16 × 10^–12^	15 × 10^–12^	14 × 10^–12^	s
τ_i_	3.8 × 10^–9^	3.6 × 10^–9^	3.4 × 10^–9^	s
τ_M_	5.5 × 10^–7^	3.6 × 10^–7^	2.3 × 10^–7^	s
*S*^2^	0.71			
τ_l_	1.7 × 10^–10^	1.2 × 10^–10^	0.8 × 10^–10^	s
ZFS	0.03			cm^–1^
θ	35	47	55	degrees

a Fixed values. The outer-sphere parameters *d* (distance of closest approach) and *D* (diffusion coefficient) were fixed to 3.6 Å and to 1.8 × 10^–9^, 2.3 × 10^–9^, and 3.0 × 10^–9^ m^2^ s^–1^ at 15, 25, and 37 °C, respectively.

The best-fit profiles were obtained by allowing the angle θ between the *z* axis of the ZFS tensor and the line passing through the positions of the gadolinium ion and the coordinated water molecule to change with changing temperature. The quality of the fit gets worse if θ is constrained to be the same at all temperatures (unless the squared order parameter *S*^2^, τ_v_, and τ_i_ are unrealistically allowed to increase with increasing temperature). Some inaccuracy in the calculated rates is expected because τ_i_ and the electron relaxation time are of the same order of magnitude for frequencies smaller than 10 MHz, and therefore the slow rotation treatment is only approximate (more general approaches than the modified Florence NMRD program have been developed^70−72^ for a more accurate treatment of these cases). This is however not preventing the accuracy of all other parameters, which are mainly determined from the relaxivity data at high fields, when ZFS is negligible and the SBM model holds.

In the fit, a single conformational state was assumed to be present. However, the variability of the angle θ with temperature may also suggest the occurrence of multiple states, with a temperature-dependent equilibrium. At low temperatures, the gadolinium chelate may in fact interact to some extent with protein residues, whereas at higher temperatures, the increased mobility of the tag can favor more free and extended conformations.

**Figure 6:**
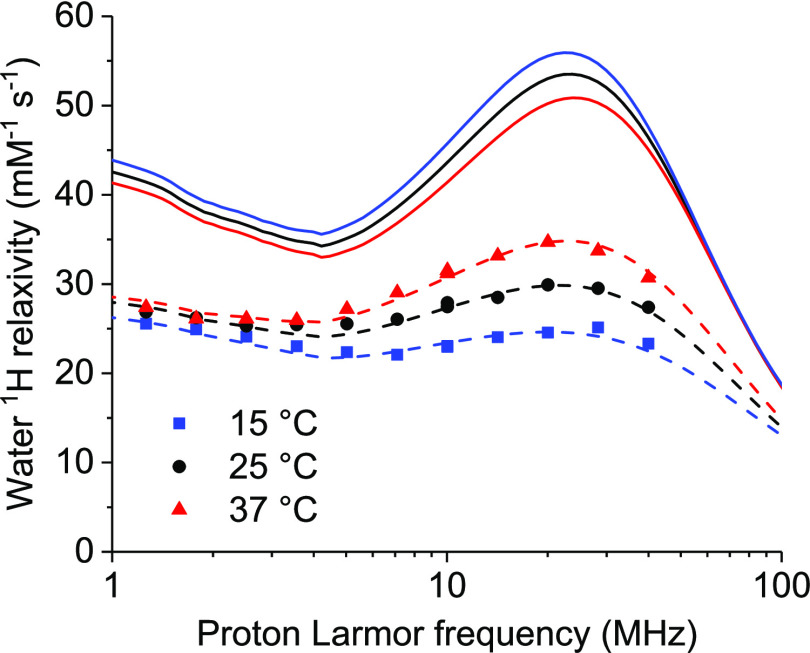
^1^H relaxivity profiles calculated for optimized water exchange (τ_M_ ≈ 2–3 × 10^–8^ s) and all other parameters equal to the values reported in Table 2 (solid lines). The experimental data for GdDOTA-conjugated ANSII are also reported.

The fit indicates an optimal reorientation time of few nanoseconds, and a lifetime of the coordinated water molecule of 0.2–0.5 μs, i.e., about double with respect to the values observed for the free chelates in water.^73,74^ A marked increase in the lifetime of the coordinated water molecule is often observed for gadolinium chelates conjugated to other proteins and macromolecular substrates. This increase is usually ascribed to the formation of hydrogen bonds involving the coordinated water itself and/or between the carboxylic groups of the ligand and amino acid side chains on the surface of the protein, which result in a significant release of the electric charge on the complex that, in turn, yields slower dissociation kinetics.^75^Figure 6 shows the largest enhancement in relaxivity, which can be achieved for an optimal lifetime of the coordinated water molecule of 20–30 ns. The profiles show that, for an optimized water exchange, at 1.5 T (ca. 60 MHz proton Larmor frequency), the relaxivity could increase from 25 to 34 s^–1^ mM^–1^. An even larger enhancement can be obtained at lower fields. An increase in the reorientation time, on the contrary, would produce a decrease in water relaxivity for fields higher than 1 T.

## Conclusions

The relaxometric profiles of the Gd-labeled ANSII indicate a relaxivity at about 1 T more than 5 times higher than that of clinically used contrast agents and quite high for a gadolinium-labeled protein. The analysis of the profiles sheds light on the origin of the observed relaxivity enhancement. The main contribution arises from the increased reorientational correlation time, amounting to few nanoseconds, i.e., only 1 order of magnitude larger than that of the unbound paramagnetic complex, but optimal for high-field MRI (1.5 T). Conversely, the observed slow exchange regime of the coordinated water molecules limits the relaxivity enhancement. It has been hypothesized that slow exchange arises from a pattern of hydrogen bonds involving the coordinated water itself, the carboxylic groups, and the amino acids at the protein surface. This view is supported by the short length of the linker connecting the paramagnetic core to the protein residues. We can speculate that the use of a longer linker may on the other hand decrease the reorientation time, leading to a decrease in relaxivity. A more interesting solution could be the use of a different gadolinium chelate, characterized by a faster exchange of the coordinated water, as, for instance, some DO3A derivatives,^19^ which are sufficiently stable and inert for biological use.^76^ The tetrameric assembly of the protein and the high relaxivity of the gadolinium chelates are key factors that make Gd(III)-labeled asparaginase an interesting model to develop new theranostic agents.

## Experimental Procedures

### Protein Expression and Purification

ANSII was expressed and purified in both nonlabeled and ^15^N-labeled forms, following the published protocol for ANSII production and purification.^77^

### Conjugation Reaction

DOTA-NHS-ester solution was prepared by dissolving the reagent in a volume of dry DMF so that the percentage of organic solvent in the final 12 mL reaction volume was less than 1%. An excess of DOTA-NHS-ester (15 times the monomer concentration) with respect to a 0.152 mmol dm^–3^ protein solution was employed for the conjugation. The reaction occurred overnight in 150 mmol dm^–3^ phosphate buffer, pH 7.5, at room temperature.

A desalting column (Hi prep 26/10) was performed to eliminate the unreacted DOTA-NHS-ester and to change the buffer to 50 mmol dm^–3^ MES–NaOH 100 mmol dm^–3^ NaCl, pH 6.5.

### Chelation Reaction

A 10 mmol dm^–3^ solution of GdCl_3_ was added to the conjugated protein solution, to have a concentration of gadolinium(III) equal to 80% of the estimated DOTA-NHS-ester bound to the protein. The solution was incubated at 309 K and under stirring for 3 days overall.

Size exclusion chromatography using a HiLoad 16/60 Superdex 75 pg column was performed to remove the nonreacted gadolinium(III) ions.

### ICP-OES

Inductively coupled plasma coupled with optical emission spectrometry was employed to determine gadolinium(III) concentration into the conjugated ANSII-DOTA sample.

### ESI MS Spectrometry

The ESI MS investigations were performed using a TripleTOF 5600+ high-resolution mass spectrometer (Sciex, Framingham, MA), equipped with a DuoSpray interface operating with an ESI probe. All of the ESI mass spectra were acquired through direct infusion at 7 μL min^–1^ flow rate.

The ESI source parameters optimized for the protein are the following:

Positive polarity, ionspray voltage floating (ISFV) 5500 V, temperature (TEM) 25 °C, ion source gas 1 (GS1) 25 L min^–1^; ion source gas 2 (GS2) 0 L min^–1^; curtain gas (CUR) 20 L min^–1^, collision energy (CE) 10 V; declustering potential (DP) 30 V, acquisition range 500–3400 *m*/*z*.

For acquisition, Analyst TF software 1.7.1 (Sciex) was used and deconvoluted spectra were obtained using the Bio Tool Kit micro-application v.2.2 embedded in PeakView software v.2.2 (Sciex).

Both samples were diluted to a final protein concentration of 10^–6^ M using LC-MS water, pH 5.5, and the 0.5% v/v of formic acid was added just before the infusion in the mass spectrometer to enhance the ionization process.

The percentages of free ANSII and of its conjugates in the final sample has been calculated according to the relative intensity of each MS peak.

### ^1^H NMRD

Nuclear magnetic relaxation dispersion (NMRD) profiles were acquired with a fast-field-cycling Stelar relaxometer. They provided the field dependence of the longitudinal relaxation rate of water protons in samples with ANSII-DOTA solutions, from 0.01 to 40 MHz proton Larmor frequency.^57^

## Supporting Information Available

Percentages of free and conjugated ANSII (Figure S1) and pKa and buried surface of amines of ANSII (Table S1) (PDF)
