# Supplementary material for: Large Protein Assemblies for High-Relaxivity Contrast Agents: The Case of Gadolinium-Labeled Asparaginase
Source: Bioconjug Chem. 2022 Dec 2;33(12):2411–9. doi: 10.1021/acs.bioconjchem.2c00506 (PMC9782335; doi:10.1021/acs.bioconjchem.2c00506)
Supplement: Supplementary file 1 — bc2c00506_si_001.pdf [file bc2c00506_si_001.pdf]

Large protein assemblies for high relaxivity contrast agents: the case of gadolinium-labeled asparaginase

Giulia Licciardi,[a,b,c] Domenico Rizzo,[a,b,c] Maria Salobehaj,[a,b,c] Lara Massai,[b] Andrea Geri,[b] Luigi Messori,[b] Enrico Ravera,[a,b,c] Marco Fragai,[a,b,c] Giacomo Parigi[a,b,c] *

[a]Magnetic Resonance Center (CERM), University of Florence, via Luigi Sacconi 6, Sesto Fiorentino, 50019 Italy; [b]Department of Chemistry "Ugo Schiff", University of Florence, via della Lastruccia 3, Sesto Fiorentino, 50019 Italy; and [c]Consorzio Interuniversitario Risonanze Magnetiche Metallo Proteine (CIRMMP), via Luigi Sacconi 6, Sesto Fiorentino, 50019 Italy

Supporting information

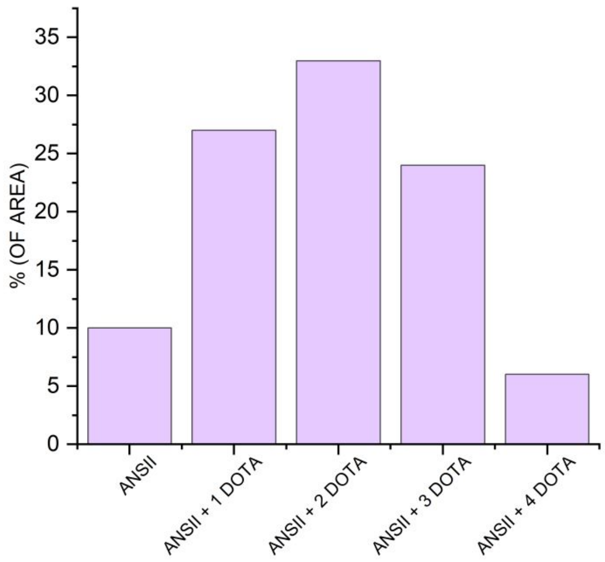

Figure S1. The percentages of free and conjugated ANSII in the sample has been calculated according to the relative intensity of each peak.

Table S1. pK_a and buried surface of amines of ANSII

	RESIDUE NUMBER	pK_a	BURIED %
**N+**	1	7.85	0
**LYS**	301	7.94	100
**LYS**	104	8.71	48
**LYS**	172	9.37	93.75
**LYS**	49	9.63	11.75
**LYS**	71	9.98	0
**LYS**	162	10.02	100
**LYS**	262	10.16	0
**LYS**	196	10.28	0
**LYS**	107	10.30	3
**LYS**	43	10.36	0
**LYS**	186	10.41	10.25
**LYS**	314	10.46	7.75
**LYS**	139	10.48	0
**LYS**	213	10.50	2.5
**LYS**	207	10.51	0
**LYS**	79	10.89	0
**LYS**	251	10.92	0
**LYS**	288	10.94	0
**LYS**	229	11.47	0
**LYS**	72	12.33	3.5
